# Mechanisms underlying AD‐sleep interactions: Recent insight from animal models

**DOI:** 10.1002/alz.090824

**Published:** 2025-01-03

**Authors:** Sigrid C Veasey

**Affiliations:** ^1^ Univ of PA TRL Room 2115, philadelphia, PA USA

## Abstract

Epidemiologic studies have revealed a relationship between chronic persistent short sleep and the risk of dementia, yet whether short sleep is an early symptom of dementia or a contributor to dementia in humans has not been resolved. A rapidly growing body of research in animal models, however, strongly supports the concept that chronic disruption of sleep can impart significant injury in the brain consistent with some of the important pathology evident in Alzheimer’s disease (AD), the most common form of age‐related dementia. Transgenic models of AD pathology develop an increased density of amyloid plaques in response to chronic sleep loss, and this response is independent of significant stress. Studies in animal models without a genetic predisposition to AD pathology have identified late‐in‐life neural injury with many features observed in AD, including increased amyloid‐beta in the hippocampus, gliosis, memory impairment and loss of AD‐vulnerable neurons. Moreover, sleep loss impairs microglial capacity to clear amyloid fibrils. The chronically sleep‐deprived brain shows a unique proteomic profile in response to sleep loss, in cerebrospinal fluid (CSF) independent of plaque. Can we now identify who is at risk for AD‐like injury from a sleep disorder by assessing CSF, or identify subtypes of AD based on CSF findings? Noradrenergic locus coeruleus neurons are susceptible to loss in AD and sleep loss, where noradrenaline reuptake and lysosomal dysfunction in the neurons may contribute to injury. We should now determine whether chronic sleep loss in humans increases specific lysosomal markers in the blood or cerebral spinal fluid, and if so, these biomarkers may be used to identify individuals at risk and for early AD. As locus coeruleus activity can now be monitored non‐invasively in humans, this work also highlights novel avenues to detect early neural injury in Alzheimer’s and assess the effectiveness of early therapies. Thus, the time is right to integrate new findings in humans and in animal models of sleep disruption to effectively advance diagnostics and therapies for AD and related dementias.

